# ccmGDB: a database for cancer cell metabolism genes

**DOI:** 10.1093/nar/gkv1128

**Published:** 2015-10-30

**Authors:** Pora Kim, Feixiong Cheng, Junfei Zhao, Zhongming Zhao

**Affiliations:** 1 Department of Biomedical Informatics, Vanderbilt University School of Medicine, Nashville, TN 37203, USA; 2 Department of Cancer Biology, Vanderbilt University School of Medicine, Nashville, TN 37232, USA; 3 Department of Psychiatry, Vanderbilt University School of Medicine, Nashville, TN 37212, USA

## Abstract

Accumulating evidence has demonstrated that rewiring of metabolism in cells is an important hallmark of cancer. The percentage of patients killed by metabolic disorder has been estimated to be 30% of the advanced-stage cancer patients. Thus, a systematic annotation of cancer cell metabolism genes is imperative. Here, we present ccmGDB (Cancer Cell Metabolism Gene DataBase), a comprehensive annotation database for cell metabolism genes in cancer, available at http://bioinfo.mc.vanderbilt.edu/ccmGDB. We assembled, curated, and integrated genetic, genomic, transcriptomic, proteomic, biological network and functional information for over 2000 cell metabolism genes in more than 30 cancer types. In total, we integrated over 260 000 somatic alterations including non-synonymous mutations, copy number variants and structural variants. We also integrated RNA-Seq data in various primary tumors, gene expression microarray data in over 1000 cancer cell lines and protein expression data. Furthermore, we constructed cancer or tissue type-specific, gene co-expression based protein interaction networks and drug-target interaction networks. Using these systematic annotations, the ccmGDB portal site provides 6 categories: gene summary, phenotypic information, somatic mutations, gene and protein expression, gene co-expression network and drug pharmacological information with a user-friendly interface for browsing and searching. ccmGDB is developed and maintained as a useful resource for the cancer research community.

## INTRODUCTION

Malignant cells exhibit specific metabolic signatures that may be linked to both genetic and epigenetic alterations (1). Many studies have demonstrated that rewiring of metabolism in cells is another general hallmark of cancer and can be used as a therapeutic target (2–4). The Warburg effect (5,6) is a good example. Under stressful metabolic conditions and hypoxic microenvironment, cancer cells react to support the needs for survival and rapid proliferation via glycolysis and metabolic pathway reprogramming (3). The importance of cell metabolism control in cancer can be estimated by the percentage of patients who are killed by a metabolic disorder called cancer-associated cachexia (CAC), rather than by the tumor itself; this percentage was estimated to be up to 30% of advanced-stage cancer patients in a previous study (7). A large volume of cancer genomic data generated from The Cancer Genome Atlas (TCGA) project indicate that somatic alterations of cell metabolism genes represent important genetic signatures that may drive tumor initiation and progression and may be related to anticancer drug responses (4). Several cell metabolism genes like *PKM* (8), *HK2* (9), *IDH1* (10) and *HIF1A* (11) have been proven to be promising targets in molecular cancer therapy. Therefore, comprehensive annotations of all cell metabolism genes may provide important resources for researchers to better understand cancer mechanisms and identify potential druggable cancer cell metabolism targets (4).

During the past decade, many studies have reported that cancer genes may mediate the reprogramming of cell metabolism. In one article, 10 cell metabolism genes were systematically reviewed for their mechanisms in oncogenesis as well as their potential as diagnostic markers and therapeutic targets (12). Metabolism and oxidative stress have been found to be connected when researchers examined the *ETS1* expression profile in ovarian and breast cancers (13). A set of cancer metabolism pathways were inferred from a list of genes overexpressed in cancer (14). Recently, the expression patterns of 1421 genes extracted from the Kyoto Encyclopedia of Genes and Genomes (KEGG) metabolic pathways were examined using microarray gene expression data (15). So far, there has not been a systematic collection and curation of cancer cell metabolism genes. With the exponential growth of cancer and other biomedical data, the demand to develop a database to systematically explore the global and specific features of cell metabolism genes in cancer has become especially urgent in the cancer research community.

In this paper, we describe ccmGDB (Cancer Cell Metabolism Gene DataBase) and its website with several applications. ccmGDB enables users to effectively browse and systematically explore the genetic, genomic, transcriptomic, proteomic and functional information of cell metabolism genes in cancer. As the first database focusing on cancer cell metabolism genes, ccmGDB provides useful information for cancer cell metabolism studies and broad biomedical research.

## DATABASE OVERVIEW

ccmGDB contains over 2000 cell metabolism genes that are annotated with 6 categories. (i) The gene summary category provides basic gene information and diverse hyperlinks for gene expression, protein annotation, ortholog information, metabolism annotation, regulation and gene context information. In addition, this category shows the manually curated articles for each cancer cell metabolism gene through manually checking over 2000 PubMed articles by our experts. (ii) The phenotypic category allows user to explore disease or phenotype related information such as the cancer gene databases including cancer cell metabolism genes (ccmGene), disease related database links and mouse phenotype database links. (iii) The somatic alteration annotation category presents different types of somatic mutations. In the current version of ccmGDB, there are 151 238 somatic nucleotide variants (SNVs), 5916 small insertions and deletions (indels), 6288 copy number variants (CNVs; 4504 copy number gain and 1784 copy number loss), and 1971 structural variants (SVs) that were extracted from COSMIC and 102 399 SNVs that were obtained from TCGA. For translocation or gene rearrangement information, we integrated 4729 human chimeric transcripts for cell metabolism genes (cmGenes) from Chitars2.0 (16). (iv) The expression category is based on the Cancer Cell Line Encyclopedia (CCLE) (17), TCGA, and The Cancer Proteome Atlas (TCPA) data and provides cell-line specific and primary cancer type specific gene expression patterns and cancer type specific protein expression patterns. For example, 78% (1632) of cmGenes had differential gene expression patterns for 8 cancer types of TCGA data. (v) The gene–gene network category provides the results for exploring different pathway activities between tumor and normal samples based on co-expressed protein interaction network derived from 113 473 protein–protein interactions. (vi) The pharmacological annotation category offers drug-centric and gene-centric networks to dynamically show the druggable features of cancer cell metabolism targets using 4059 drugs. Furthermore, ccmGDB offers a cross-referenced ID table, which is primarily based on parsed Universal Protein Resource (Uniprot) data (18).

Table 1 summarizes the statistics for cmGenes and ccmGenes per each annotation category. The current database includes 2071 cmGenes and 514 ccmGenes. Almost all of these genes have mutation and gene expression information derived from COSMIC and TCGA. Furthermore, ccmGDB includes 946 drug related cmGenes, 1392 cmGenes having translocations and approximately 1500 unannotated cmGenes that are not well-studied in cancer. Such data can be used to explore and predict cancerous features and possible drug repurposing. All aforementioned entries and annotation data are available to browse and search on the ccmGDB website.

**Table 1.** Annotation entry statistics for all cell metabolism genes

Data type	# Entries	# cmGenes^a^	# ccmGenes^b^
		Total 2071 (%)	Total 514 (%)
Cancer genes	# genes		
Oncogenes^c^	41	41 (2.0%)	41 (8.0%)
Tumor suppressor genes^d^	92	92 (4.4%)	92 (17.9%)
Cancer Gene Census^e^	50	50 (2.4%)	50 (9.7%)
Cancer genes^f^	382	382 (18.4%)	382 (74.3%)
Network of cancer genes^g^	133	133 (6.4%)	133 (25.9%)
Significantly mutated driver genes^h^	110	110 (5.3%)	110 (21.4%)
Pathway	# pathways (# genes)		
KEGG^i^	42 (922)	922 (44.5%)	210 (40.9%)
REACTOME^j^	27 (1597)	1597 (77.1%)	406 (79.0%)
Interaction^k^	# interactions		
Physical interaction^l^	679 507	1968 (95.0%)	481 (93.6%)
Metabolic interaction^m^	21 353	1149 (55.5%)	245 (47.7%)
Signaling interaction^n^	78 548	1131 (54.6%)	361 (70.2%)
Expression	# samples		
CCLE^o^	1037	1893 (91.4%)	488 (95.0%)
TCGA^p^	4150 (tumor)	2061 (99.5%)	514 (100%)
	461 (normal)		
RPPA^q^	4775	24 (1.2%)	21 (4.1%)
Mutation	# mutations		
TCGA^r^	102 399 SNVs^s^	2026 (97.8%)	508 (98.8%)
COSMIC^t^	151 238 SNVs	2040 (98.5%)	510 (99.2%)
	5916 Indels^u^	1213 (58.5%)	340 (66.1%)
	6288 CNVs^v^	1836 (88.6%)	461 (90.0%)
	1971 SVs^w^	782 (37.7%)	225 (43.8%)
Chitars2.0^x^	4729 chimeric transcripts	1392 (67.2%)	392 (76.3%)
Molecule	# molecules		
DrugBank^y^	4059 drugs	946 (45.7%)	269 (52.3%)
UniProt^z^	2062 proteins	2069 (99.9%)	514 (100%)

^a^Cell metabolism genes.

^b^Cancer cell metabolism genes.

^c^Oncogenes from Cancer Genes.

^d^Tumor suppressors from TSGene.

^e^Cancer genes from Census of human cancer genes.

^f^Cancer genes from CancerGenes. ^g^Cancer genes from NCG4.0.

^h^Significantly mutated genes per 18 TCGA cancer types from 12 articles.

^i^Cell metabolism related pathway in KEGG.

^j^Cell metabolism related pathway in REACTOME.

^k^PathwayCommons interaction.

^l^Genes having ‘interacts-with’, ‘reacts-with and neighbor-of’ interactions among PathwayCommons.

^m^Genes having ‘catalysis-precedes’ interactions among PathwayCommons.

^n^Genes having ‘controls-production-of’, ‘in-complex-with’, ‘controls-state-change-of’, ‘controls-phosphorylation-of’, ‘controls-transport-of’, ‘controls-expression-of’, ‘consumption-controlled-by’, ‘controls-transport-of-chemical’ and ‘chemical-affects’ interactions among PathwayCommons.

^o^Gene expression for cancer cell lines of 24 cancer types.

^p^RNA-seq data for primary tumor and normal samples.

^q^Protein expression values.

^r^Mutations called for TCGA exome-seq data by TCGA investigators.

^s^Somatic nucleotide variations.

^t^All types of variants collected in COSMIC.

^u^Insertions and/or deletions.

^v^Copy number variations.

^w^Structural variants.

^x^Human chimeric transcripts.

^y^Related drug with the cmGene.

^z^Universal protein ID for the cmGene.

## DATA INTEGRATION

### Cell metabolism genes

**Figure 1.**
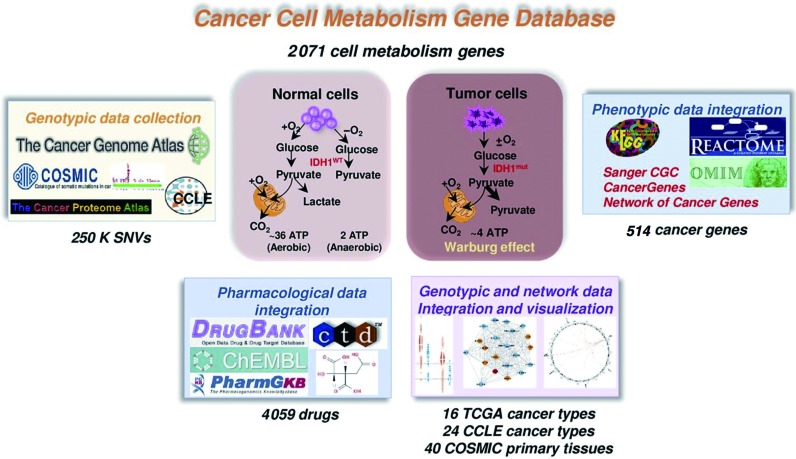
Overview of ccmGDB. Cancer cell metabolism gene database is composed of 6 categorized annotations from the integration of genotypic data, phenotypic data, pharmacological data and network analysis for all 2071 cell metabolism genes.

Figure 1 shows an overview of ccmGDB. The current version includes 2071 cell metabolism genes that were collected from 42 KEGG (19) and 27 REACTOME (a knowledgebase of biological pathways) (20) metabolic pathways. These KEGG and REACTOME pathways included 922 and 1597 genes, respectively.

### Annotation of cancer cell metabolism genes

We integrated cancer gene information from five different cancer gene databases: Oncogene (21), TSGene (22), Cancer Gene Census (CGC) (23), CancerGenes (21) and Network of Cancer Genes (NCG4.0) (24). This integration strategy is to annotate the well-studied metabolic targets for cancer therapy based on a previous review article (4). We further included cancer type-specific significantly mutated genes from over 20 TCGA genome analysis projects and other published data (25–41). Through gene ID mapping with all cmGenes, we extracted 514 ccmGenes. As a result, the ccmGenes data set is composed of 41 Oncogenes, 92 TSGenes, 50 CGC genes, 382 CancerGenes, 133 Network of Cancer Genes and 110 significantly mutated genes. In addition, 689 genes had candidate metabolic therapeutic vulnerabilities based on homozygous deletions (42). Specifically, we found three common genes among the five cancer gene sets in the KEGG cell metabolic pathway: *PTEN, AKT1* and *PIK3CA*. The detailed information is shown in Supplementary Figure S1.

### Manual curation of articles showing cancer cell metabolism genes’ function

For 514 cancer cell metabolism genes and 10 important metabolic genes not included in ccmGenes, we performed a literature query of PubMed in September, 2015, using the search expression that applied to each ccmGene (using *IDH1* as an example here): ‘((cancer cell metabolism [Title/Abstract]) AND *IDH1*[Title/Abstract]) AND (‘2001/01/01’[Date - Publication] : ‘2015/09’[Date - Publication])’. From these abstracts, we manually checked over 2000 articles. We found 242 genes (∼47%) having literature evidence (492 articles), supporting the function of these genes by regulating cell metabolism in cancer. Using this curation, we created a classification system to introduce reliability. Class A requires literature evidence and belonging to the cancer gene. Class B requires only belonging to the cancer gene and the other genes belong to Class C.

### Mutation data integration

Somatic point mutations were collected from TCGA (March, 2014). In addition, we collected point mutations, indels, CNVs and SVs from the COSMIC v72 data sets of GRCh37. To find more translocation or gene rearrangement information, we downloaded 20 750 human chimeric transcripts from Chitars2.0 (16) data and compared these with cmGenes. Among them, 4729 chimeric transcripts were related with 1392 cmGenes. In addition, we downloaded CNV data from TCGA (January, 2015) and extracted them using the R package in TCGA-Assembler. Using the ProcessCNAData function in the TCGA-Assembler package, we obtained the gene-level CNV data calculated as the average copy number of the genomic region of each gene.

### Expression data preparation

We downloaded gene expression data from TCGA (January, 2015). Normalized gene expression data from RNASeqV2 were extracted using the R package TCGA-Assembler (43). In addition, microarray gene expression data in over 1000 cancer cell lines was extracted from CCLE (October, 2012) using gene-centric RMA-normalized mRNA expression data. Differential gene expression visualization was done using the beanplot package in R. Reverse Phase Protein Array (RPPA) data were extracted from TCPA (44). Normalized values based on replicate-based normalization (RBN) were used to draw images. A total of 4032 images about gene expression were included in the ccmGDB database.

### Co-expressed protein interaction network (CePIN)

We used 113 473 unique protein–protein interactions connecting 13 579 protein-coding genes to construct a protein interaction network (PIN) as done in our previous study (45,46). We then calculated the Pearson Correlation Coefficient (PCC) for each gene–gene pair using the RNASeqV2 data and mapped the PCC value of each gene–gene pair onto the above PIN to build a CePIN based on two previous studies (45,47). Co-expressed network figures were drawn using the igraph package in R. For each gene, the top 20 neighbors having the highest PCC values were used in the network. The selection of 20 neighbors reflects the genetic signals while controlling the subnetworks so as not to be too large. The target gene was labeled in red while other cancer cell metabolism genes in the same network were marked in orange.

### Drug–gene interaction network

We extracted drug-target interactions (DTIs) from three resources: DrugBank (48), the Therapeutic Target Database (TTD) (49) and the PharmGKB database (50). Drugs were grouped using Anatomical Therapeutic Chemical (ATC) classification system codes (51). All genes encoding drug targets were mapped to their Entrez IDs based on the National Center for Biotechnology Information (NCBI) database (52). Duplicated DTI pairs were excluded. All chemical two-dimensional structural images of drugs were generated using the chemical toolbox, OpenBabel (v2.3.1) (53).

### Database architecture

The ccmGDB system is based on a three-tier architecture: client, server and database. It includes a user-friendly web interface, Perl's DBI module and MySQL database. The database of ccmGDB was developed on MySQL 3.23 with the MyISAM storage engine.

## WEB INTERFACE AND APPLICATIONS

### Somatic mutation category

**Figure 2.**
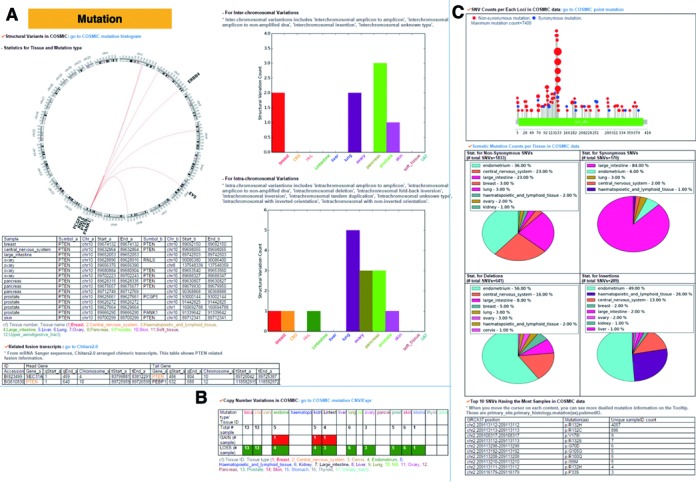
Mutation category in ccmGDB. (**A**) Structural variants annotation for *PTEN*. A Circos plot based on chromosomes and detailed information including cancer type specific statistics and fusion gene information is provided. (**B**) Copy number variations annotation for *PTEN*. Copy number gain is colored in red and copy number loss in green. (**C**) Somatic single nucleotide variations and small insertions and deletions for *IDH1* such as mutation frequency per tissue and protein structure based representation.

The mutation category presents SNVs, indels, CNVs and SVs with cancer type-specific and sub categorized mutation type-specific information, as shown in Figure 2. The SV part supports genomic rearrangements and structural variants related information using the data for 12 tissue types from COSMIC. This information includes Circos plots and tables for inter-chromosomal and intra-chromosomal rearrangements per tissue type as shown in Figure 2A. Through integration and comparison with the database of human chimeric transcripts and RNA-sequencing (Chitars2.0), we could get 4729 chimeric transcripts for cmGenes. The CNV part gives copy number variation information for 16 cancer types from TCGA and variation types (GAIN or LOSS). Figure 2B shows the copy number loss of tumor suppressor gene *PTEN* in 10 cancer tissues. SNV information part includes SNV loci and frequency information at amino-acid sequence, SNV counts, percentage per cancer type and the top 10 SNVs in the highest recurrence, as shown in Figure 2C. The isocitrate dehydrogenase 1 gene (*IDH1*)'s mostly frequently observed non-synonymous SNV is a well-known driver mutation (R132H) in the central nervous system (81.0%) (Figure 2A), which is consistent with a previous study (54).

### Gene expression category

**Figure 3.**
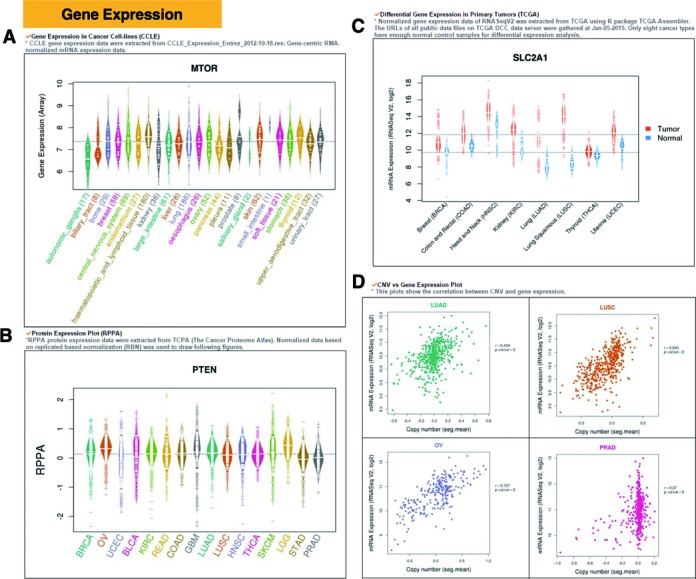
Expression category in ccmGDB. Using this category, user can compare the expression level per cancer/tissue type at a glance. (**A**) Gene expression plot of *mTOR* for cancer cell lines using CCLE data. (**B**) Protein expression plot of *PTEN* using TCPA data. (**C**) Differential gene expression plot of *SLC2A1* for primary cancer tissues using TCGA data. (**D**) Correlation plot between gene expression and copy number of *mTOR* for TCGA data.

This category includes cancer/tissue type-specific gene expression, differential gene expression, protein expression and the correlation between gene expression and CNVs. Figure 3A shows an example of cell line-specific expression in 24 cancer types from CCLE for *mTOR* (encoding mammalian target of rapamycin) which is a critically deregulated gene in the cell-signaling pathway in various human cancer types (55). In addition, ccmGDB provides phosphorylated protein expression plots using the RPPA data from TCPA (44). One example is shown in Figure 3B for activated *PTEN* expression in ovarian serous cystadenocarcinoma (OV) and lower grade glioma (LGG) (56). Differential gene expression analyses for eight cancer types of TCGA were also included in ccmGDB. Among all the 2071 cmGenes and all the 514 ccmGenes, on average 1454 and 380 genes displayed differential expression patterns (adjusted *P*-value < 0.05, t-test with correction by Benjamini–Hochberg's false discovery rate (FDR)), respectively, as shown in Supplementary Table S1. Almost 50% of ccmGenes and cmGenes showed differentially expressed patterns with up- or down-regulated features. For example, *SLC2A1*, encoding a major glucose transporter in the mammalian blood-brain barrier, plays a crucial role in cancer cell metabolism (57). Figure 3C indicates that *SLC2A1* is highly expressed in all the eight tumor types compared to the matched normal samples (adjusted *P*-value < 0.05, t-test with correction by Benjamini–Hochberg's FDR). In addition, ccmGDB provides a correlation analysis between gene expression and CNVs. Figure 3D shows that *mTOR* is highly amplified in lung squamous cell carcinoma (LUSC) with a positive correlation with CNVs among 15 different TCGA cancer types.

### Gene–gene network category

**Figure 4.**
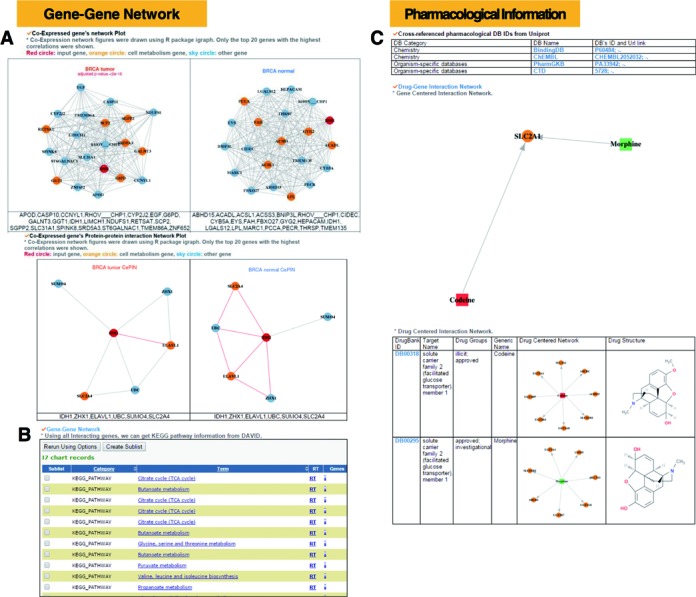
Gene–gene network category and pharmacological category. (**A**) Co-expressed protein interaction network using the top 20 co-expressed genes for *IDH1*. By gene set enrichment analysis (GSEA) of the 20 genes with the cancer/tissue type-specific information in this category, user can infer differentially activated pathways. The target gene is colored in red and other cmGenes in orange. (**B**) Enriched KEGG pathway information using all interacting genes from PathwayCommons. (**C**) Pharmacological information for *SLC2A1*. Gene-centric network, drug-centric networks and detailed information for each drug including the two-dimensional structure information are provided.

The gene–gene network category provides cancer/tissue type-specific co-expressed gene network and co-expressed protein interaction network (CePIN) analysis for the top 20 co-expressed genes having the highest gene–gene co-expression correlation for each cmGene across 8 cancer types and normal tissues as shown in Figure 4A. Using this annotation, we performed a gene set enrichment analysis for *IDH1* (Supplementary Table S2) with WEB-based Gene SeT AnaLysis Toolkit (WebGestalt) (58). The top enriched pathway in BRCA was ‘carbohydrate metabolic process’ with *q*-value 0.0019, which corresponds to ‘glycolysis’. The ‘*NADPH* regeneration’ pathway was also significantly enriched in breast cancer samples with *q*-value 0.0041. The ‘*NADPH* regeneration’ pathway has a major role in the pentose phosphate pathway (PPP), ATP formation pathway via glycolysis. On the other hand, the normal samples’ enriched pathways showed energy transduction processes via oxidative phosphorylation. These results would suggest to us the possibility of an energy metabolism process alteration from oxidative phosphorylation to glycolysis during tumorigenesis. In addition, ccmGDB displays meaningful KEGG pathway information for each target gene via a popular bioinformatics tool DAVID (59) using all the interacting genes from PathwayCommons data as shown in Figure 4B.

### Drug pharmacological category

Figure 4C shows a drug–gene network visualization using both gene-centric and drug-centric fashions. From a gene-centric network, user can retrieve drug names related with the target gene. From a drug centric network, user can obtain more detailed information for those drugs including DrugBank ID, target domain name, the drug's approved status, other genes related with this drug and the two-dimensional drug structure. We identified potential druggable genes targeting tumor metabolism through constructing a drug-target interaction subnetwork connecting 80 approved or experimental drugs and 23 significantly mutated cell metabolism genes. Supplementary Figure S3 shows several druggable targets that are significantly mutated in cancer, such as *AKT1, PIK3CA, MTOR, IDH1 and PIK3R1*. We found that several known anticancer drugs can regulate cancer cell metabolism pathways, such as caldribine, sirolimus, everolimus, temsirolimus and imatinib. Cladribine was approved for the treatment of chronic lymphocytic leukemia and cutaneous T-cell lymphoma (48). However, the exact mechanism-of-action (MOA) of cladribine for cancer treatment is unknown. Supplementary Figure S3 indicates that cladribine targets a significantly mutated cancer gene *POLE*, which is a key DNA repair gene. A previous cancer genome study reported that *POLE* is significantly mutated in uterine cancer (36) and this gene was specifically highlighted in a pan-cancer mutation signature analysis (60). Budesonide was an approved glucocorticoid agent for the treatment of allergic rhinitis (61). Supplementary Figure S3 reveals that budesonide might target *PIK3CA, PIK3R1* and *AKT1* by regulating cell metabolism activity. Previous preclinical and clinical studies showed that budesonide is a very promising agent for lung cancer chemoprevention (62,63).

## DISCUSSION AND FUTURE DIRECTION

This study presents a unique resource, ccmGDB, for the systematic annotation of cell metabolism genes in cancer. Among 2071 cell metabolism genes, 77% have not been deeply studied in cancer yet. Using ccmGDB, user can search cancer-related genetic, genomic, transcriptomic, proteomic, functional information and systematic somatic mutation annotations. However, more detailed annotations for regulation such as microRNA, epigenetic alterations and other gene regulation information have not been systematically done. Previous studies have reported that microRNAs and epigenetic changes also play critical roles in cancer cell metabolism (64); thus, we plan to annotate such data in the near future. Furthermore, there are several methods to quantitate metabolites like Consumption and Release (CORE) profiling (65) and Metabolic Flux Analysis (MFA) (66). We anticipate an increasing number of metabolite quantitation studies in the next a few years. If so, we will integrate these data in ccmGDB as well.

To serve cancer cell metabolism researchers for the development of novel targeted cancer therapy, we will continuously update ccmGDB and provide a unique resource in the following directions. (i) Collect high-quality microRNA data that regulate cell metabolism in the particular cancer type and add microRNA–gene regulation information (67). We will expand this effort to include other types of non-coding RNA such as long non-coding RNA (lncRNA) too. (ii) Add more comprehensive cancer genetic and genomic data, including methylation, and regulatory profiles of non-coding somatic mutation data from several whole-genome sequencing and functional genomic projects, such as the NIH Roadmap Epigenetics (68) and the International Cancer Genome Consortium (ICGC) (69) projects. (iii) Add more high-quality drug pharmacological data from high-throughput screening studies and drug resistance studies for more positive clinical outcome and better therapeutics.
